# Synergistic suppression of the PI3K inhibitor CAL-101 with bortezomib on mantle cell lymphoma growth

**DOI:** 10.7497/j.issn.2095-3941.2015.0013

**Published:** 2015-12

**Authors:** Fu-Lian Qu, Bing Xia, Su-Xia Li, Chen Tian, Hong-Liang Yang, Qian Li, Ya-Fei Wang, Yong Yu, Yi-Zhuo Zhang

**Affiliations:** ^1^Department of Hematology, Tianjin Medical University Cancer Institute and Hospital, National Clinical Research Center for Cancer, Tianjin Key Laboratory of Cancer Prevention and Therapy, Tianjin 300060, China; ^2^Department of Medical Oncology, Kaifeng Central Hospital, Kaifeng 475000, China; ^3^Department of Geriatric Hematology, Chinese PLA General Hospital, Beijing 100853, China

**Keywords:** CAL-101, bortezomib (BTZ), phosphatidylinositol-3-kinase (PI3K), mantle cell lymphoma (MCL)

## Abstract

**Objective:**

To investigate the effects of CAL-101, particularly when combined with bortezomib (BTZ) on mantle cell lymphoma (MCL) cells, and to explore its relative mechanisms.

**Methods:**

MTT assay was applied to detect the inhibitory effects of different concentrations of CAL-101. MCL cells were divided into four groups: control group, CAL-101 group, BTZ group, and CAL-101/BTZ group. The expression of PI3K-p110σ, AKT, ERK, p-AKT and p-ERK were detected by Western blot. The apoptosis rates of CAL-101 group, BTZ group, and combination group were detected by flow cytometry. The location changes of nuclear factor kappa-B (NF-κB) of 4 groups was investigated by NF-κB Kit exploring. Western blot was applied to detect the levels of caspase-3 and the phosphorylation of AKT in different groups.

**Results:**

CAL-101 dose- and time-dependently induced reduction in MCL cell viability. CAL-101 combined with BTZ enhanced the reduction in cell viability and apoptosis. Western blot analysis showed that CAL-101 significantly blocked the PI3K/AKT and ERK signaling pathway in MCL cells. The combination therapy contributed to the inactivation of NF-κB and AKT in MCL cell lines. However, cleaved caspase-3 was up-regulated after combined treatment.

**Conclusion:**

Our study showed that PI3K/p110σ is a novel therapeutic target in MCL, and the underlying mechanism could be the blocking of the PI3K/AKT and ERK signaling pathways. These findings provided a basis for clinical evaluation of CAL-101 and a rationale for its application in combination therapy, particularly with BTZ.

## Introduction

Mantle cell lymphoma (MCL), an aggressive disease that accounts for 5%-10% of all lymphomas, is incurable with conventional therapies^1^^-^^3^. The prevalence of MCL has been increasing over the last few decades, particularly among elderly patients^4^^,^^5^. Despite various therapeutic options, MCL remains an incurable disease with a median survival time of less than 3 years^6^.

Phosphatidylinositol-3-kinases (PI3Ks) are enzymes that mediate signals from cell surface receptors. Class I PI3K isozymes (PI3Kα, PI3Kβ, PI3Kγ, and PI3Kδ) regulate various cellular functions by producing phosphatidylinositol-3,4,5-triphosphate^7^^,^^8^. Generation of phosphatidylinositol-3,4, 5-triphosphate activates downstream serine/threonine kinases, namely, Akt and mammalian target of rapamycin (mTOR), which positively affects cell survival, proliferation, growth, and metabolism^9^^,^^10^. Dysregulation of the PI3K/Akt/mTOR pathway is important in the etiology of human malignancies^11^. Of several PI3K isoforms, PI3Kδ plays a pivotal function in B-cell signaling in response to chemokines and cytokines. CAL-101, also named GS-1101, is a novel oral PI3Kδ-specific inhibitor that demonstrates activity in other types of B-cell cancers^12^^-^^14^. In this study, we characterized the effect of CAL-101 on MCL cells to provide translational support for PI3Kδ inhibition as a novel strategy for MCL treatment.

Bortezomib (BTZ), a boronic anhydride that functions as a reversible proteasome inhibitor, was the first clinically available drug and approved for refractory multiple myeloma (MM)^15^. BTZ preferentially targets malignant cells^16^, and the mechanisms underlying the proteasome inhibitor lethality of BTZ include misfolded protein accumulation, endoplasmic reticulum stress induction, oxidative injury, nuclear factor kappa-B (NF-κB) inhibition (by IκBα accumulation), pro-apoptotic protein up-regulation, p53 and JNK stabilization, and DNA repair interference. Although several factors support the strategy of combining CAL-101 with proteasome inhibitors in non-Hodgkin’s lymphoma (NHL), including MCL, the effect of CAL-101 when combined with BTZ on MCL cells remains unknown. Therefore, we aim to clarify whether a synergistic effect exists between CAL-101 and BTZ in MCL.

## Methods

### Cells and reagents

The human MCL cell lines, namely, Z138, HBL-2, and Jeko-1, were provided by the Moffitt Cancer Center. These cells were cultured in RPMI-1640 medium supplemented with 10% fetal bovine serum (Hyclone), 1% (v/v) penicillin (100 U/mL), and streptomycin (100 U/mL) at 37 °C in an incubator with 5% CO_2_ under standard humidity. The specific proteasome inhibitor BTZ (also named Velcade and formerly known as PS-341) was provided by the Department of Hematology and Bone Marrow Transplantation of Tianjin Medical University Cancer Institute and Hospital. CAL-101 was purchased from Selleck Company (Catalog No. S2226). CAL-101 was dissolved in dimethyl sulfoxide (DMSO) as a stock solution, stored at 80 °C, and diluted with serum-free RPMI 1640 prior to use. The final DMSO concentration should not exceed 0.1% for all experiments.

### Cell viability assay

Cell viability was quantified using MTT assay. Z138, HBL2, and Jeko-1 cells were seeded in 96-well plates (8,000 cells/well) and incubated for 0, 24, 48, and 72 h with different CAL-101 concentrations (0, 20, 40, 60, 80, and 90 μmol/L) with or without BTZ (0, 0.06, 0.12, 0.18, 0.24, and 0.3 μmol/L). The cells were added with MTT reagent (Sigma-Aldrich) and then incubated at 37 °C for 3 h. The solution was decanted, and 100 μL of DMSO was added to dissolve purple formazan crystals. The absorbance of the resulting solution was determined at 570 nm with a microplate reader (Synergy 2, Bio Tek, VT, USA). Each dose was tested in triplicate. Dose effect and combination index (CI) curves were constructed using the CalcuSyn software. Data were confirmed by performing at least three independent experiments.

### Apoptosis analysis

About 5×10^5^ Z138 and HBL2 cells were cultured in six-well plates. The cells were exposed to four groups: CAL-101 group (45 μmol/L CAL-101), BTZ group (0.3 μmol/L BTZ), CAL-101/BTZ combination group (45 μmol/L CAL-101 and 0.3 μmol/L BTZ), and control group (no drug added but supplement with RPMI-1640 medium) for 48 h. After treatment, the cells were washed with PBS and suspended in 100 µL of 1× binding buffer. Annexin V-FITC (5 μL) and PI (5 μL) were added. After the cells were incubated at room temperature for 15 min in dark, apoptosis rate was assessed with FACS (Becton Dickinson, USA). Data were analyzed using the FlowJo 2.7.4 software program (Tree Star, USA).

### NF-κB activity

About 5×10^5^ Z138 and HBL2 cells were cultured in six-well plates. The cells were exposed to four groups: CAL-101 group (45 μmol/L CAL-101), BTZ group (0.3 μmol/L BTZ), CAL-101/BTZ combination group (45 μmol/L CAL-101 and 0.3 μmol/L BTZ), and control group (no drug added but supplemented with RPMI-1640 medium) for 48 h. After the cells were treated, nuclear protein was extracted using a nuclear extraction kit (Active Motif, Carlsbad, CA, USA). NF-κB activity was determined using the TransAM NF-κB p65 transcription factor assay kit (Active Motif) according to the manufacturer’s instruction.

### Western blot analysis

About 5×10^5^ Z138, HBL2, and Jeko-1 cells were cultured in six-well plates and treated with various CAL-101 concentrations (0, 10, 20, and 40 μmol/L) for 48 h. In combination experiments, the cells were divided into four groups: CAL-101 group (45 μmol/L CAL-101), BTZ group (0.3 μmol/L BTZ), CAL-101/BTZ combination group (45 μmol/L CAL-101 and 0.3 μmol/L), and control group (no drug added but supplemented with RPMI-1640 medium) for 48 h. Proteins were extracted from the MCL cell lines and analyzed through SDS-PAGE. Immunoblot analysis was performed using antibodies against p-ERK, p-AKT, AKT, ERK, caspase-3 (Cell Signaling Technology), and β-actin (Abcam, USA).

### Statistical analysis

The SPSS19.0 software program was used for statistical analysis. Results were presented as mean ± standard deviation (SD) and examined for significant differences using Student’s *t* test. An alpha value of *P*<0.05 was considered statistically significant difference. Synergistic drug interactions were tested through median dose-effect analysis with a commercially available software program (CalcuSyn, Biosoft, MO).

## Results

### CAL-101 and BTZ synergistically suppressed MCL cell viability

**Figure 1:**
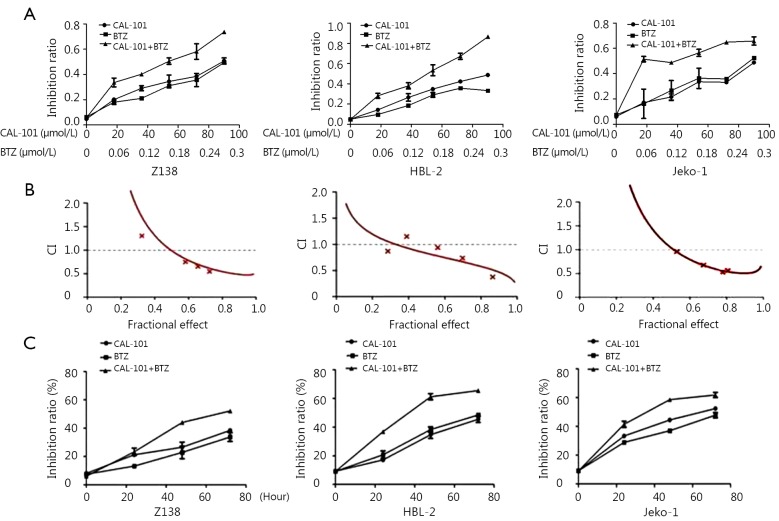
CAL-101 and bortezomib synergistically interact in Z138, HBL-2, and Jeko-1 cells. (A) CAL-101/bortezomib combination significantly suppressed viability of MCL cells compared with single-agent CAL-101 or BTZ through MTT. (B) CAL-101/bortezomib combination induced significantly synergistic cytotoxicity in MCL cells by CalcuSyn software calculation [Combination index (CI) <1.0]. CI values <1.0 indicates a synergistic interaction and CI values >1.0 indicates antagonistic drug effects. A straight line at CI =1.0 represents additive effects of both drugs. (C) Inhibitory effect of CAL-101 on the viability of Z138, HBL-2, and Jeko-1 cells was time dependent.

The cytotoxic effects of single-agent CAL-101 or BTZ were studied, and IC50 was evaluated in three different MCL cell lines through MTT assay. The IC50 of single-agent CAL-101 and BTZ were 56.96 and 0.335 µmol/L in Z138 cells, respectively, 62.99 and 0.46 µmol/L in HBL-2 cells, respectively, and 77.82 and 0.388 µmol/L in Jeko-1 cells, respectively (**Figure 1A**). To determine the effect of combined exposure to both agents on cell viability, we incubated the cells with various concentrations of CAL-101 and BTZ for 48 h. Fractional effect-CI curves were constructed (**Figure 1B**). The CI values of CAL-101-BTZ were lower than 1 for the three cell lines, which demonstrated the synergistic inhibition of the drug combination. Furthermore, CAL-101 exhibited a time-dependent inhibitory effect on the viability of Z138, HBL-2, and Jeko-1 cells. CAL-101 and BTZ concentrations were fixed at 60 and 0.18 μmol/L, respectively (**Figure 1C**).

### Co-administration of CAL-101 and BTZ synergistically enhanced apoptosis in MCL cells

**Figure 2:**
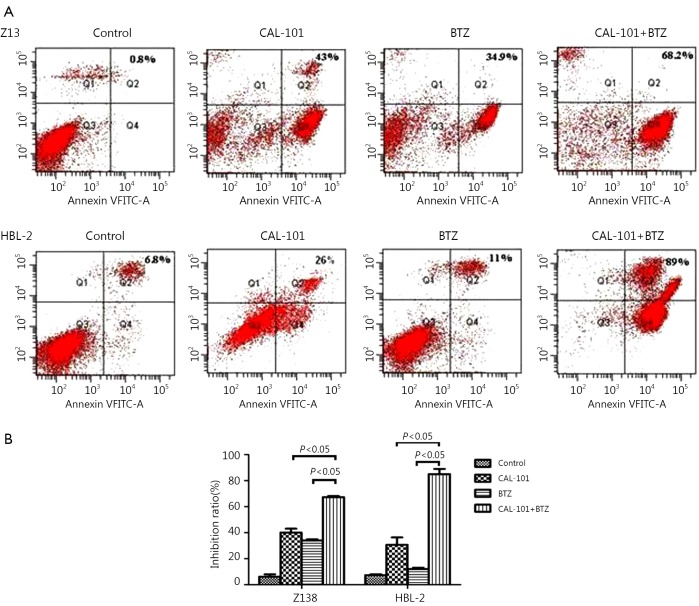
Combined treatment with CAL-101 and bortezomib synergistically induces apoptosis in MCL cells. (A) Annexin-V/PI assay indicated that CAL-101/BTZ combination obviously induced cell apoptosis in two cell lines. (B) Bar chart showed that the combined treatment produced enhanced apoptosis in both cell lines.

To determine the effect of combined exposure to both agents on apoptosis, we treated the cells with CAL-101 with or without BTZ for 48 h. Annexin staining for apoptosis was then performed. The result showed that treatment with CAL-101 and BTZ resulted in a pronounced increase in apoptosis in Z138 and HBL-2 cells (**Figure 2**). The results of dose-response studies further revealed that cell exposure to 0.3 µmol/L BTZ and 45 µmol/L CAL-101 for 48 h resulted in significant increase in cell death. These findings suggested that combined treatment of CAL-101 and BTZ potently induced apoptosis in MCL cell lines.

### CAL-101 inhibited the PI3K/AKT and ERK pathways in MCL cells

**Figure 3:**
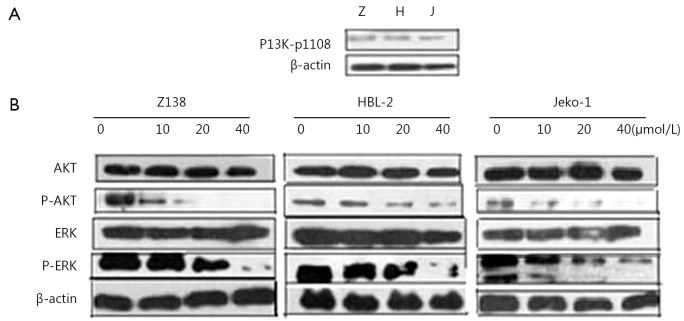
CAL-101 inhibits the PI3K/AKT and ERK pathway. (A) PI3K-p110σ was expressed in MCL cell lines. (B) The expression of P-AKT and P-ERK decreased with increasing concentration of CAL-101.

We demonstrated that PI3K-p110σ was expressed in Z138, HBL-2, and Jeko-1 cells (**Figure 3A**). After the cells were treated with CAL-101, AKT and ERK phosphorylation were dose-dependently inhibited (**Figure 3B**). This finding indicated that PI3K/AKT and ERK signaling was inhibited.

### Co-exposure of MCL cells to CAL-101 and BTZ inactivated the NF-κB and AKT pathways

**Figure 4:**
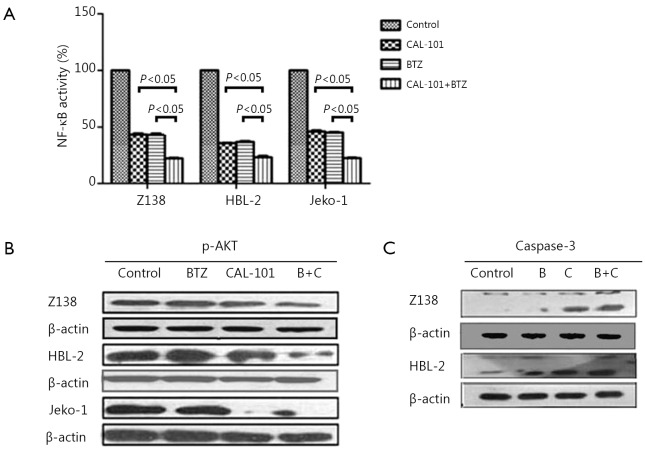
(A) NF-κB Inactivation is enhanced by CAL-101/Bortezomib combination. (B) Combined treatment induced marked downregulation of p-AKT in MCL cells. (C) Caspase-3 was up-regulated after combined treatment in MCL cell lines.

The effects of the combination of CAL-101 and BTZ on NF-κB activation were assessed in Z138, HBL-2, and Jeko-1 cells by using an ELISA TransAM NF-κB p65 transcription factor assay kit (Active Motif). NF-κB activity was significantly less after combined exposure to both agents than that after exposure to single agent in Z138, HBL-2, and Jeko-1 cells. This finding suggested that the combination of CAL-101 and BTZ enhanced NF-κB inactivation (**Figure 4A**). We demonstrated that PI3K/AKT signaling may also be inhibited by CAL-101. The cells were treated with the combined treatment, and the results showed the induced p-AKT down-regulation in Z138, HBL-2, and Jeko-1 cells (**Figure 4B**). Cleaved caspase-3, which is the downstream kinase of the AKT pathway, was up-regulated after the combined treatment (**Figure 4C**).

## Discussion

MCL, a distinct subset of B-cell NHL, is characterized by the t(11;14)(q13;q32) chromosomal translocation. Several survival pathways involving NF-κB, phosphatidylinositol-4, and PI3K have been reported to be associated with MCL^17^^,^^18^. Conventional treatment with immunochemotherapy and autologous stem cell transplantation has shown improved outcomes, and recent advances in MCL treatment are promising.

CAL-101, a selective inhibitor of PI3K/p110σ, exhibits excellent clinical activity in CLL, diffuse large B cell lymphoma, and MM^19^^-^^21^. The PI3K survival pathways are deregulated in several malignancies, including MCL. The inhibition of the PI3K pathway by CAL-101 (GS-1101, idelalisib) blocks survival signals, induces apoptosis, and disrupts signals from the tumor microenvironment, resulting in B-cell malignancies.

CAL-101 exhibits cytotoxic activity on MCL cells. Previous studies demonstrated that AKT is constitutively activated in an MCL subset^22^^-^^27^. AKT activation promotes cell survival, proliferation, and apoptosis inhibition through multiple mechanisms, including NF-κB activation^28^^-^^30^. As CAL-101 is a PI3K inhibitor, we then tested PI3K activity after treatment with CAL-101. We found that CAL-101 inhibited AKT and ERK phosphorylation (p-AKT and p-ERK), which demonstrated that CAL-101 cytotoxicity in MCL cells was mediated via an inhibitory effect on the PI3K/AKT pathway and ERK signaling. This finding supported that p110σ, p-AKT, and p-ERK were involved in cell growth and proliferation.

BTZ, a reversible proteasome inhibitor, was approved for refractory MCL, but this drug has limited single-agent capacity^31^^-^^34^. The AKT and ERK pathways are important for malignant cell survival^35^, and interruption of each pathway potentiates proteasome inhibitor lethality in cancer cells^36^^,^^37^. These findings supported the feasibility of using the combination of CAL-101 and BTZ for MCL. We found that CAL-101 and BTZ synergistically induced cell death in MCL cells, induced inhibition of AKT and NF-κB activity, and significantly enhanced cell apoptosis.

In summary, we reported that CAL-101 was cytotoxic to human MCL cells and potently enhanced the activity of the proteasome inhibitor BTZ. The underlying mechanism could be the targeting of NF-κB and AKT activity. In line with the emerging or established evidence of the activity of these agents in MCL, this regimen must be further investigated *in vivo*. Overall, we provide a basis for clinical trials of CAL-101 in MCL and a rationale for its use in combination therapy with BTZ.
